# Mindfulness as Medicine: Why Internal Medicine Must Embrace Psychological Health

**DOI:** 10.7759/cureus.96521

**Published:** 2025-11-10

**Authors:** Taimoor Jaffar Ali

**Affiliations:** 1 Internal Medicine, King Edward Medical University, Lahore, PAK

**Keywords:** internal medicine, mind-body medicine, mindfulness, mindfulness-based interventions, physician well-being, psychological health, stress physiology

## Abstract

Psychological health exerts a profound influence on physical disease, yet it remains an underappreciated dimension within internal medicine. Emerging evidence demonstrates that chronic stress, depression, and anxiety can alter cardiovascular, endocrine, and immune pathways, contributing to poorer clinical outcomes. Mindfulness, defined as the intentional awareness of the present moment with acceptance and without judgment, offers a scientifically validated means of addressing these mind-body interactions. Research on mindfulness-based interventions, including Mindfulness-Based Stress Reduction and Mindfulness-Based Cognitive Therapy, has shown improvements in blood pressure, glycemic control, pain modulation, and overall well-being. Internists, who manage chronic illness and long-term patient relationships, are uniquely positioned to integrate mindfulness into clinical care through brief in-office practices, structured referrals, and stress screening tools. Beyond patient benefit, mindfulness supports physician empathy, resilience, and reduced burnout. Reimagining internal medicine through the lens of mindfulness restores its humanistic essence and unites the biological and psychological dimensions of healing. The next revolution in internal medicine may not come from a molecule but from a moment of mindful awareness.

## Editorial

Introduction

In clinical practice, physicians frequently observe that patients with similar pathophysiological conditions respond differently to identical treatments. Among these variations, one of the most consistent determinants of outcomes is psychological health. Numerous studies have demonstrated that emotional distress, depression, and anxiety significantly worsen cardiovascular, metabolic, and inflammatory diseases. Patients experiencing chronic stress exhibit higher levels of cortisol, impaired immune regulation, and slower recovery after illness [1]. Despite this growing body of evidence, psychological well-being remains a peripheral consideration in most internal medicine settings. The discipline has traditionally emphasized objective measurements, biochemical markers, and imaging findings, often overlooking the patient’s mental landscape [2]. This reductionist approach has contributed to fragmented care that addresses disease but not the individual experiencing it. Internal medicine must now evolve to embrace psychological health as an integral part of healing, recognizing that the mind and body operate as one interconnected system rather than separate domains of care.

The psychophysiology of illness

The intricate relationship between emotional states and physical health is firmly established through extensive empirical and mechanistic evidence. Chronic psychological stress activates the hypothalamic-pituitary-adrenal (HPA) axis, leading to sustained elevations of cortisol and catecholamines. This dysregulated neuroendocrine activity promotes endothelial dysfunction, impairs glucose metabolism through insulin resistance, and suppresses immune surveillance [2,3]. Prolonged exposure to these mediators creates a proinflammatory environment that accelerates atherosclerosis, disrupts metabolic equilibrium, and delays tissue repair. Mood disorders such as depression and anxiety further intensify these disturbances. Elevated levels of C-reactive protein, interleukin-6, and tumor necrosis factor-alpha exemplify the inflammatory signature of psychological distress. Concurrently, chronic stress reduces heart rate variability, an established marker of autonomic imbalance, predisposing individuals to arrhythmias, hypertension, and other adverse cardiovascular outcomes.

Findings from psychoneuroimmunology confirm that these changes are neither transient nor benign but exert a cumulative influence on disease onset, progression, and recovery. In patients with coronary artery disease, heightened psychological stress independently predicts poorer post-revascularization outcomes [3,4]. Similarly, individuals with diabetes show worsened glycemic control during periods of psychological distress, underscoring the bidirectional relationship between mind and body [5]. Evidence suggests that mindfulness-based interventions can mitigate these biological disruptions by lowering cortisol levels, stabilizing glycemic control, reducing systemic inflammation, and improving blood pressure regulation. Psychological well-being, therefore, is not peripheral but a measurable determinant of physical health. The body reflects the mind, and the mind continually shapes the body’s physiology.

From complementary to core clinical practice

Mindfulness is a structured and intentional state of awareness in which attention is directed toward the present moment with acceptance and without judgment. In clinical science, it is recognized as a therapeutic construct grounded in cognitive, behavioral, and neurobiological mechanisms rather than a spiritual tradition. Evidence demonstrates that it modulates neural circuits responsible for attention, emotion regulation, and interoception, particularly within the prefrontal cortex, amygdala, and insula. These neural effects correspond with physiological improvements, including reduced sympathetic activation, enhanced parasympathetic tone, and normalization of HPA axis function.

Mindfulness-Based Stress Reduction (MBSR) and Mindfulness-Based Cognitive Therapy (MBCT) are among the most extensively studied frameworks and are now regarded as evidence-based interventions across diverse medical conditions [2,3]. In hypertension, they lower blood pressure through improved autonomic regulation and vascular reactivity; in type 2 diabetes, they enhance dietary adherence, glycemic control, and metabolic stability. In chronic pain and irritable bowel syndrome, these interventions mitigate symptoms by altering cortical pain processing and reducing visceral hypersensitivity [5]. Beyond symptom relief, they foster patient engagement, strengthen adherence to treatment plans, and reduce healthcare utilization. The convergence of neurobiological, behavioral, and clinical evidence affirms mindfulness as a core element of internal medicine, bridging the physiological and psychological dimensions of healing and reinforcing a model of care that treats the patient as an integrated whole [4].

Healing the healer and the patient

Internists play a central role in long-term patient care, making them well-positioned to address both biological and psychological dimensions of illness. Integrating mindfulness into internal medicine transforms conventional biomedical encounters into holistic experiences that honor the patient as a whole person. Even brief guided practices during consultations, structured referrals to MBSR programs, and the use of validated stress-screening tools can yield meaningful clinical benefits [1,3]. Evidence also highlights its profound effect on physicians, who experience reduced emotional exhaustion, greater empathy, and improved self-regulation. Neuroimaging studies demonstrate enhanced activation of brain regions governing emotional regulation and perspective-taking, correlating with more compassionate, patient-centered communication. When both physician and patient engage in mindfulness-informed care, the clinical encounter evolves from a transactional exchange into a shared process of awareness, trust, and mutual healing, preserving the emotional resilience and humanity of the healer [2].

Normalizing psychological care within internal medicine

Despite a robust and expanding evidence base, the incorporation of mindfulness into internal medicine remains inconsistent and limited. Several barriers continue to impede its integration into mainstream clinical practice. Chief among these is the enduring perception that mindfulness belongs to the domain of “soft science,” lacking the empirical rigor traditionally associated with biomedical disciplines [1]. Additional challenges include insufficient exposure during medical education, limited time within outpatient workflows, and the absence of standardized reimbursement mechanisms for mindfulness-based interventions. These systemic and cultural obstacles collectively sustain a clinical environment that undervalues psychological determinants of health.

Overcoming these barriers requires deliberate educational and institutional reform. Residency curricula should include structured modules on the psychophysiology of stress, the neurobiology of mindfulness, and practical training in evidence-based mindfulness programs. This would equip future internists with the competence to integrate psychological assessment and mindfulness-informed techniques into routine care. In clinical settings, brief three- to five-minute mindfulness protocols can be seamlessly incorporated into patient encounters, enhancing therapeutic presence without compromising efficiency. Institutional endorsement by academic bodies, medical boards, and professional associations is equally essential to ensure credibility and sustainability. When internal medicine formally acknowledges psychological well-being as a quantifiable and clinically relevant dimension of health, it will realign itself with the foundational principle of holistic, person-centered medicine-care that encompasses both the measurable and the meaningful aspects of human well-being [4,5].

The humanistic imperative

Medicine exists at the intersection of empirical science and humanistic practice. While its scientific dimension demands precision, procedural skill, and adherence to evidence-based standards, its humanistic essence is grounded in empathy, moral responsibility, and authentic presence. Effective healing requires physicians to treat not only biological pathology but also the patient’s emotional and existential realities, fulfilling the ethical duty of beneficence that emphasizes care for the person rather than the disease [2]. Mindfulness bridges the science and art of medicine by cultivating attention, emotional attunement, and compassion. Through this awareness, physicians engage more deeply with patients, strengthening diagnostic accuracy, therapeutic alliance, and relational trust. Neurocognitive evidence shows that mindfulness enhances neural activity in regions associated with empathy and emotion regulation, affirming its biological and ethical foundations [3]. Far from being a philosophical abstraction, mindfulness represents a clinical competency that rehumanizes medical practice, transforming care from a procedural act into a shared experience of understanding and trust, where scientific rigor and compassion coexist in service of the whole patient.

A call to mindful medicine

Internal medicine is entering a defining era in which the integration of psychological health is no longer optional but fundamental to its continued scientific and ethical advancement [1]. The convergence of neuroscience, behavioral medicine, and clinical outcomes research has made it clear that psychological well-being exerts measurable influence over nearly every physiological system. Mindfulness offers a rigorously studied and ethically grounded framework through which internal medicine can bridge the historical divide between body and mind. By cultivating awareness, presence, and compassion, physicians not only enhance therapeutic efficacy and patient satisfaction but also safeguard their own professional resilience and moral integrity. Embracing mindfulness does not represent a departure from biomedical progress; rather, it signifies its natural maturation - a synthesis of empirical knowledge with humanistic wisdom. The next revolution in internal medicine may not come from a molecule but from a moment of mindful awareness [2].
